# HIV Viral Suppression among People Living with HIV on Antiretroviral Therapy in Haut-Katanga and Kinshasa Provinces of Democratic Republic of Congo

**DOI:** 10.3390/healthcare10010069

**Published:** 2021-12-31

**Authors:** Gulzar H. Shah, Lievain Maluantesa, Gina D. Etheredge, Kristie C. Waterfield, Osaremhen Ikhile, Roger Beni, Elodie Engetele, Astrid Mulenga

**Affiliations:** 1 Jiann-Ping Hsu College of Public Health, Georgia Southern University, Statesboro, GA 30460, USA; oi00156@georgiasouthern.edu; 2 FHI 360 Congo, Kinshasa, Congo; LMaluantesa@fhi360.org (L.M.); EEngetele@fhi360.org (E.E.); AMulenda@fhi360.org (A.M.); 3 FHI 360, Washington, DC 20009, USA; GEtheredge@fhi360.org; 4 Department of Interdisciplinary Healthcare, University of North Georgia, Dahlonega, GA 30597, USA; kristie.waterfield@ung.edu; 5 National AIDS Control Program (PNLS), HIV Program, Ministry of Health, Kinshasa, Congo; beniroger2002@yahoo.fr

**Keywords:** HIV viral load suppression, antiretroviral therapy, Democratic Republic of Congo, TB/HIV co-infection, WHO clinical stages

## Abstract

Human immunodeficiency virus (HIV) infections and less-than-optimal care of people living with HIV (PLHIV) continue to challenge public health and clinical care organizations in the communities that are most impacted by HIV. In the era of evidence-based public health, it is imperative to monitor viral load (VL) in PLHIV according to global and national guidelines and assess the factors associated with variation in VL levels. Purpose: This study had two objectives—(a) to describe the levels of HIV VL in persons on antiretroviral therapy (ART), and (b) to analyze the significance of variation in VL by patients’ demographic and clinical characteristics, outcomes of HIV care, and geographic characteristics of HIV care facilities. Methods: The study population for this quantitative study was 49,460 PLHIV in the Democratic Republic of Congo (DRC) receiving ART from 241 CDC-funded HIV/AIDS clinics in the Haut-Katanga and Kinshasa provinces of the DRC. Analysis of variance (ANOVA) was performed, including Tamhane’s T2 test for pairwise comparisons using de-identified data on all patients enrolled in the system by the time the data were extracted for this study by the HIV programs in May 2019. Results: The VL was undetectable (<40 copies/mL) for 56.4% of the patients and 24.7% had VL between 40 copies/mL and less than 1000 copies per mL, indicating that overall, 81% had VL < 1000 and were virologically suppressed. The remaining 19% had a VL of 1000 copies/mL or higher. The mean VL was significantly (*p* < 0.001) higher for males than for females (32,446 copies/mL vs. 20,786, respectively), persons <15 years of age compared to persons of ages ≥ 15 years at the time of starting ART (45,753 vs. 21,457, respectively), patients who died (125,086 vs. 22,090), those who were lost to follow-up (LTFU) (69,882 vs. 20,018), those with tuberculosis (TB) co-infection (64,383 vs. 24,090), and those who received care from urban clinics (mean VL = 25,236) compared to rural (mean VL = 3291) or semi-rural (mean VL = 26,180) clinics compared to urban. WHO clinical stages and duration on ART were not statistically significant at *p* ≤ 0.05 in this cohort. Conclusions: The VL was >1000 copies/mL for 19% of PLHIV receiving ART, indicating that these CDC-funded clinics and programs in the Haut-Katanga and Kinshasa provinces of DRC have more work to do. Strategically designed innovations in services are desirable, with customized approaches targeting PLHIV who are younger, male, those LTFU, with HIV/TB co-infection, and those receiving care from urban clinics.

## 1. Introduction

With the advancement of technology and science, HIV has transitioned from a life-threatening terminal illness to a manageable chronic disease. In order for patients to have an overall good prognosis, HIV prevention programs have to assess and address the severity of infection by relying on regular measurements of viral load (VL), which is described as the number of copies of HIV RNA in a milliliter of blood [1]. VL suppression is the most important milestone on the continuum of HIV care in the era of undetectable equals untransmittable (U = U) [2]. According to the Centers for Disease Control and Prevention (CDC) data, an HIV-positive patient is considered to be virally suppressed and at “effectively no risk” of transmission to an HIV-negative person when the VL is considered “durably undetectable” and remains undetectable for at least six months after the first undetectable result [3,4]. Both the CDC and the World Health Organization (WHO) recognize and promote the benefits of ART for VL suppression in PLHIV [5,6]. The WHO also recommends that VL monitoring be regularly conducted after PLHIV are placed on ART, with first the VL test at six months after ART initiation and annual testing thereafter [7].

To eradicate HIV/AIDS by 2030, the Joint United Nations Program on HIV and AIDS (UNAIDS) developed goals that focus on diagnosis, therapy, and viral suppression. UNAIDS has set 95–95–95 targets to be met by the year 2030 [8]. Globally in 2019, data show that only 31% of PLHIV that are receiving ART are virally suppressed. In the western and central regions of Africa, the viral suppression rate is 45% and varies greatly by country [9]. According to the 2020 UNAIDS Report, the Democratic Republic of Congo (DRC) had achieved 54% of the first 95 (percentage of PLHIV who know their status), and 98% of the second 95 (percentage of PLHIV that know their status who are on ART). However, the data regarding PLHIV who are on ART and have suppressed VL (third 95) were not available [9]. 

As HIV programs continue to prioritize their efforts in increasing the number of PLHIV that are receiving ART and subsequently have VL suppression, there is mounting clinical evidence that increases the recognition that there are many genetic and behavioral factors that can affect how a person may respond to therapy. Some of these factors include the age and gender of the patient, whether the patient continues to seek care, disease progression before ART is initiated, and adherence to the therapy protocol. According to the WHO and the CDC, the primary determining factor of VL suppression and risk of transmission is the patient’s adherence to ART. Unfortunately, long-term adherence in low- and middle-income countries (such as the DRC) is met with numerous patient- and program-related challenges, such as forgetting to take medication, low health literacy, substance abuse, long wait times at clinics, distance to clinics, and costs of care [7,10]. Although adherence to ART directly influences viral suppression, some patients with long-term adherence to ART are also experiencing viral rebound due to either suboptimal adherence after a period of good adherence or ART-resistant mutations [11,12]. ART interruption is also known as interruption in treatment (IIT). IIT is another critical issue for HIV prevention and management programs because it not only increases the risk of HIV spread, but PLHIV experiencing IIT also tend to have poorer outcomes, such as a higher risk of mortality compared to those that have not experienced IIT [13]. 

Previous studies have shown that there are differences in VL based on gender; women have been shown to have significantly lower VL than men, and the differences have been attributable to hormone levels and plasma HIV RNA thresholds [14,15,16,17]. Additional studies have also shown that children (18 months to 18 years of age) were more likely to reach viral suppression than adults, which can be attributed to children being less likely to experience IIT because they often visit vaccination clinics, their growth may be monitored regularly, and parents may continue to bring children to clinics for treatment [18,19]. The studies also reported that older adults (65 years and older) were more likely to achieve VL suppression than younger adults [2,18,20]. However, the gender and age differences diminish at later stages of the disease [15,16,17,21,22]. Thus, researchers have recommended that providers ensure that ART is being administered appropriately based on gender, age, and CD4 counts [14,15,16].

Efficient program planning and strategic priority-setting in HIV clinical care programs require systematic assessment and evaluation of the outcomes in the populations served by the programs. An important objective of such data-driven assessments is to generate evidence of change in HIV patient outcomes and to assess the positive impact of clinical services over time. To that end, this study had two objectives—to examine the levels of HIV VL in persons on ART, and to analyze factors associated with variation in VL, including patients’ demographic and clinical characteristics, outcomes of HIV care such as death and LTFU, and rurality–urbanicity of HIV care clinics. In the DRC, the HIV programs are mainly funded by the U.S. President’s Emergency Plan for AIDS Relief (PEPFAR) and the Global Fund. The national HIV/AIDS program coordinates HIV service activities.

## 2. Materials and Methods

### 2.1. Data

This study utilized secondary data received from the National HIV/AIDS Program (PNLS). The PNLS managed the HIV clinics with the support of CDC/PEPFAR implementing partners. The study population for this quantitative study was 49,460 PLHIV receiving ART from 241 CDC-funded HIV/AIDS clinics in the Haut-Katanga and Kinshasa provinces of the DRC. The de-identified dataset comprised all patients enrolled in these 241 HIV programs at the time of data extraction in May 2019, with ART initiation dates from 2014 to 2019. The clinical database of HIV counseling, testing, and service delivery is maintained for these clinics using a national electronic records system for patient management, TIER.Net (accessed on 21 June 2021). The original data were collected under local IRB approval No. ESP/CE/229/2019 by FHI 360. Georgia Southern University (protocol number HI 9260) exempted this research from a full IRB review.

### 2.2. Dependent Variable

**Figure 1:**
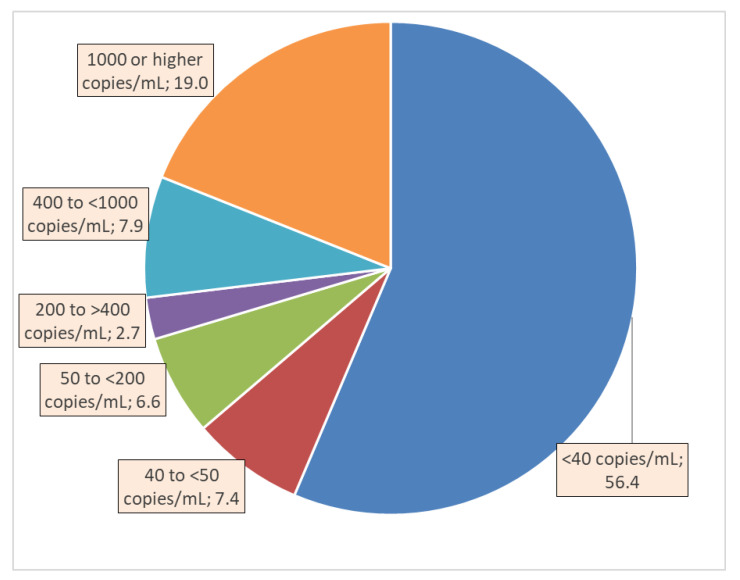
Percent distribution of persons on antiretroviral therapy by viral load levels in HIV/AIDS clinics of Haut-Katanga and Kinshasa provinces, Democratic Republic of Congo, 2014–2019.

The dependent variable *viral load* (VL) was recorded in the database as the number of copies of HIV RNA in a milliliter of blood at the time of the last VL test. This continuous variable was used as the primary outcome measure for its univariate associations with independent variables. In addition, for the graphical depiction of the VL in ranges of interest to HIV programs, the following ranges were computed (as displayed in Figure 1): <40 copies/mL, 40 to <50 copies, 50 to <200 copies, 200 to <400 copies, 400 to <1000 copies, and 1000 or more copies/mL of blood [9,12].

### 2.3. Independent Variables

The dichotomous variable *TB/HIV co-infection* was coded as “TB present” and “TB not present,” whereas records with codes 0 and 1 were excluded from our analysis. The variable *patient outcome death* was coded as 1 “died” and 2 “in care, transferred out, or LTFU.” The variable *WHO Stages* had four attributes: stage 1 (asymptomatic), stage 2 (mildly symptomatic), stage 3 (moderately symptomatic), and stage 4 (severely symptomatic). The variable *ART initiation mode* had two categories: “new patient” and “transferred in.” The variable *duration on ART* was computed by computing the time difference between “date of the last visit for ART” and “ART start date.” For our analysis, the variable *duration on ART* was recoded into quartiles, with the following categories: “<3.23 months,” “3.23 to 14.52 months,” “14.53 to 40.37 months,” and “>40.37 months.” Two demographic variables were *patient sex*, with the categories “male” and “female,” and *age at the time of the start of ART*, with the attributes shown in Table 1. *Province of health facility location* (Haut-Katanga and Kinshasa) and *rurality/urbanicity status* of the health zone were the two geographic variables. The variable *rurality/urbanicity* consisted of three categories based on the health zone—“rural,” “semi-rural,” and “urban,” as identified by the population density in cities or towns within the zone. 

### 2.4. Analytical Methods

We computed descriptive statistics—frequencies and percentages—for the dependent variable and all independent variables. To assess the associations of categorical independent variables with the VL as the continuous dependent variable, we performed analysis of variance (ANOVA). We also performed multiple comparison tests for independent variables with more than two categories if the *p*-value for ANOVA was ≤0.05, indicating that at least one pair of categories for an independent variable differed significantly in mean VL. The pairwise multiple comparisons were included in the analysis to identify which pairs of categories of each independent variable had significantly different mean VL. Because the assumption of equal variance was violated during the ANOVA test, the pairwise comparisons were based on the statistics of Tamhane’s T2 [23]. The outcome variable was found to be normally distributed, and equality of variance was assumed based upon the results of Leven’s test. All analyses for this study were performed using IBM SPSS Statistics version 25.0 (IBM Corporation, Armonk, NY, USA) [24].

## 3. Results

The VL in 56.4% of the patients was undetectable (i.e., <40 copies/mL), whereas 19.0% had the heaviest VL of 1000 copies or higher count per mL (Figure 1). The VL was 40 to <50 copies/mL for 7.4%, 50 to <200 copies/mL for 6.6%, 200 to <400 copies/mL for 2.7%, and 400 to <1000 copies/mL for 7.9% of the patients. 

Descriptive statistics for patients receiving ART at HIV/AIDS clinics (Table 1) show that 69.0% were female, and 89.7% were 15 years or older. Death was the outcome for 5.8% of patients, as opposed to 94.2% who remained in care, were transferred out, or had therapy interruption (LTFU). After excluding those who died, 16.6% were LTFU, as opposed to 83.4% who remained in care or were transferred out. TB/HIV co-infection existed in 3.6% of the patients. The highest proportion of patients, 41.9%, were in the WHO’s stage 1 of disease progression, whereas 22.9%, 30.7%, and 4.4% were in stages 2, 3, and 4, respectively. Most of the patients initiating ART therapy (95.2%) were new patients. Other trends in patient characteristics are displayed in Table 1. 

The results of the one-way ANOVA test performed to compare the mean VL by patient characteristic (Table 2) show that mean VL was significantly higher for males than for females (32,446 copies/mL vs. 20,786; *p* < 0.001, respectively). The mean VL was significantly higher in patients less than 15 years of age (45,753 vs. 21,457; *p* < 0.001). The age–sex interaction variable had a significant association with VL (*p* < 0.001); specific multiple comparisons for this and other non-dichotomous variables are discussed in the next paragraph. Patients who died had a much higher VL than those still alive (125,086 vs. 22,090; *p* < 0.001, respectively). The mean VL for patients who were LTFU was significantly higher than for those in care or transferred out (69,882 vs. 20,018, *p* < 0.001, respectively). Patients with TB co-infection also had a significantly higher mean VL of 64,383 copies per mL compared to 24,090 in those without TB co-infection (*p* < 0.001). A statistically significantly higher (*p* < 0.001) mean VL existed in patients from Kinshasa compared to those from Haut-Katanga (31,541 vs. 8021, respectively). Mean VL significantly differed by rurality levels of the health zones, with a mean VL of 3291 for rural health zones and 25,236 for urban (*p* < 0.001). WHO clinical stages and ART were not statistically significant in this cohort. 

Multiple comparison analysis for independent variables with more than two categories (Table 3) tested the significance of mean VL differences across multiple attributes of those independent variables. Post-hoc comparison using Tamhane indicated that the mean VL for <15-year-old males was significantly higher than for males 15 years or older (mean difference (MD), 22,080 copies per mL) and females 15 years or older (MD, 31,636 copies per mL). The mean VL for <15-year-old females was also significantly higher than females 15 years or older (MD, 22,593 copies per mL). Although urban vs. semi-rural facilities did not differ significantly, urban vs. rural (MD, 21,945) and semi-rural vs. rural (MD, 22,890) location of clinics showed statistically significant differences in mean VL (*p* < 0.001).

## 4. Discussion

This research aimed to assess the HIV VL and factors associated with variation in VL among PLHIV using data from the DRC’s National HIV/AIDS Program (PNLS). Our study findings regarding VL distribution will assist HIV prevention and therapy programs in the DRC to predict prognosis, classify disease progression, assess therapy success, and evaluate the risk of HIV transmission to others. In our study population, the last tested VL was undetectable (<40 copies/mL) for the majority (56.4%) of the patients, whereas roughly one-quarter (24.7%) had a VL between 40 copies/mL and less than 1000 copied per mL. Since 19% had a VL of 1000 or higher, this indicates that cumulatively, 81% had a VL < 1000 and were virologically suppressed. Other recent studies in Sub-Saharan Africa countries, such as Rwanda and Malawi, showed that up to 88% to 91% of persons on ART had a VL < 1000 [25,26]. In our study, a relatively lower proportion of PLHIV with a suppressed VL may indicate therapy gaps, which would call for evaluations by the HIV programs in the DRC to strategically revisit their therapy and coordinated services. Yet, the differences shown could be attributable to a different mix of study samples with respect to factors that can affect VL.

Our study findings on the distribution of patients by the WHO clinical stages showed that 30.7% of patients were in stage 3 and 4.4% were in stage 4. Because PLHIV at these moderately to severely symptomatic stages are at much higher risk of poor outcomes, our findings of variation in VL by specific WHO stages may inform any therapy customization effort for specific disease progression stages.

Our findings of significantly higher VL for patients who were younger than 15 years of age at the start of ART may mean that onset and diagnosis of the infection at an early age may result in greater difficulties in managing the disease. A more in-depth future study may provide specific research evidence. Our study also showed that mean VL was significantly higher for males than for females, which is consistent with the existing body of literature, and is thought to be due to differences in hormone levels, plasma and HIV RNA thresholds, and lower likelihood to access health services, meaning that males may be diagnosed later in their disease progression [14,15,16,17]. However, it may also be because, in the DRC, women have more points of entry to care and are more likely to seek care. Thus, their familiarity with more service points providing care may also improve their chances of early referrals to HIV prevention and management programs. We also noted that males who were on ART before age 15 had much higher VL compared to males or females in other age groups, potentially pointing to a synergetic effect of early-onset/diagnosis and being a male. Our study also showed that there was no association between the duration of ART and VL, perhaps due to the complexity of this relationship. Although adherence to ART is known to improve VL, the risk of therapy interruptions increases with longer duration on ART, particularly in resource-challenged environments, which can be associated with patient- and/or program-related challenges [7,10]. Our analysis of mean VL by the interaction of age at the start of ART and duration in ART showed that the worst VL levels existed in patients who started ART at ages <15 years and had been on ART for >40.37 months, a difference of 32,762 copies/mL compared with those who had started ART when they were 15 years old or older and the ART duration was 3.23 to 14.52 months. Unfortunately, patients who began ART some time ago may have started with a less effective regimen, thus allowing their disease to progress to more advanced stages before the availability of new, more effective regimens.

Our results show that therapy interruption (LTFU) has severely negative consequences for HIV VL management, calling for special attention from HIV programs to aim to improve their patient retention performance. Our findings indicate that the arithmetic mean VL for those in care or transferred out to another care facility was 20,018 copies per mL, a stark contrast to the mean VL for those with therapy interruption indicated by LTFU (69,882 copies per mL). Therefore, the strategies to reduce the IIT must be proactively and collaboratively devised by HIV prevention and management programs in order to prevent poor health outcomes such as death and/or the risk of HIV transmission [13,27]. One strategy to reduce the risk of IIT could be maintaining interoperable patient information systems, ongoing data sharing across health facilities, and ongoing data linkage to detect the patients who are LTFU at a facility but seek treatment at another facility. Another strategy is to implement decentralized drug distribution (DDD) models where ART patients can receive their medication refills in a community setting instead of at the health clinic. Multi-month dispensing may also increase the chances of retention in care, because compliant patients do not need to return often to the facility for medication pick-up. Data linkage with other social services databases may also reduce the risk of IIT. In a recent cohort study, interventions that included longer periods between ART refills (i.e., multi-month dispensation), home delivery of ART refills, linkage to social service support programs after diagnosis, and “silent” transfer of care between clinics for patients relocating (e.g., due to change in employment) were all possible successful strategies for increasing retention in care and preventing the potential for therapy interruption [28].

We found that the mean VL was significantly lower for patients seeking care at rural clinics than semi-rural or urban locations. Urban patients also have more options and may move more frequently to other clinics, interrupting their care and being listed as lost to follow-up in the absence of a unique identifier in clinical HIV care systems. Lower levels of VL in rural areas may be primarily due to the patterns of care-seeking by the severity of illness, with the sickest rural residents seeking care in urban areas. Even in semi-rural areas, the trend is for the sickest patients to move to urban areas with better-equipped and better-staffed facilities. Rural communities are often more closely knit, so the social stigma often associated with HIV may motivate the sickest rural residents to seek HIV care from urban centers away from their communities. On the other hand, in rural locations, the patients tend to have closer relationships with their healthcare providers, and patients may therefore have better adherence to healthcare providers’ instructions.

This quantitative study has some limitations; therefore, our findings should be interpreted within the context of these limitations. First, the secondary data did not contain repeated measures of the VL. In the absence of these data, VL from the last virological test was used. Were all measures of VL available for the period of study, it would have been beneficial to analyze patterns of VL changes and rebounds. Secondly, our choice of variables was limited to a small number of variables captured in the program data and shared with the research team. There are many socio-economic and lifestyle measures not captured in the dataset, which may explain the variation in VL. Finally, our data did not include information concerning the history of a patient having received enhanced adherence counseling, which may have influenced therapy adherence and, in turn, VL levels. In addition, data for other comorbidities were also not available, meriting a suggestion that future studies should also focus on the comorbidity profile of the patients. Regardless of these limitations, our findings are generalizable to HIV services in the DRC.

## 5. Conclusions

Evidence-based continuous quality improvement is increasingly deemed essential for public health services, including HIV prevention, as is management services being efficient in the face of increasing demands and shrinking resources [29]. The current study produced practice-relevant research evidence showing that although 81% of the PLHIV who were on ART had their VL suppressed below <1000 copies/mL, the clinics and programs offering these services in the Haut-Katanga and Kinshasa provinces of the DRC have more work to do. Strategically designed innovations in services, with better collaborations with community partners for referrals, are desirable to come closer to the target of 95% of all people receiving ART to have viral suppression and working towards the elimination of the AIDS epidemic. Our research findings concerning risk for un-suppressed VL suggest that innovation in the efficient delivery of services to all PLHIV is even more essential during the COVID-19 pandemic and in the post-pandemic era, because some public health and healthcare resources have been reallocated to address the emergent COVID-19 care needs, and in some settings, layering of chronic non-communicable services is becoming necessary to deal with resource shortages [29]. 

## Figures and Tables

**Table 1:** Descriptive statistics for characteristics of patients in HIV/AIDS clinics of Haut-Katanga and Kinshasa provinces, DRC, 2014–2019.

Patient Characteristics	Number	Percent (%)
Sex		
Male	15,326	31.0
Female	32,134	69.0
Age at the Time of Start of ART		
<15 years of age	4769	10.3
15 years or older	41,454	89.7
Age and Sex		
<15 years, male	2243	4.9
<15 years, female	2526	5.5
15 years or older, male	12,126	26.2
15 years or older, female	29,328	63.4
Patient Outcome—Death		
In care, transferred out or LTFU	46,609	94.2
Died	2851	5.8
Patient Outcome—LTFU		
In care or transferred out	38,872	83.4
LTFU	7737	16.6
TB Status		
No TB	43,218	96.4
TB present	1631	3.6
WHO Stage		
Stage 1	18,515	41.9
Stage 2	10,116	22.9
Stage 3	13,565	30.7
Stage 4	1960	4.4
ART Initiation Mode		
New patient	44,060	95.2
Transferred in	2214	4.8
Duration on ART in Months		
Less than 3.23 months	11,566	25.0
3.23 to 14.52 months	11,540	25.0
14.53 to 40.37 months	11,549	25.0
More than 40.37 months	11,542	25.0
Province		
Haut-Katanga	14,596	29.5
Kinshasa	34,864	70.5
Rurality/Urbanicity Status		
Rural	2790	5.6
Semi-rural	5780	11.7
Urban	40890	82.7

Abbreviations: TB, Tuberculosis; WHO, World Health Organization; ART, antiretroviral therapy; LTFU, lost to follow-up.

**Table 2:** Analysis of variance (ANOVA) results for the association of characteristics of patients with viral load in HIV/AIDS clinics of Haut-Katanga and Kinshasa provinces, DRC, 2014–2019.

Patient Characteristics	Number	Mean	95% CI		*p*
			Lower	Upper	
Sex					**<0.001**
Male	4395	32,446	26,234	38,658	
Female	10,738	20,786	17,955	23,617	
Age at the Time of Start of ART					**<0.001**
<15 years of age	1694	45,753	36,270	55,237	
15 years or older	13,436	21,457	18,663	24,252	
Age and Sex					**<0.001**
<15 years, male	798	50,536	36,954	64,119	
<15 years, female	896	41,494	28,240	54,747	
15 years or older, male	3594	28,457	21,488	35,425	
15 years or older, female	9842	18,901	16,059	21,743	
Patient Outcome—Death					**<0.001**
In care, transferred out, or LTFU	14,827	22,090	19,428	24,752	
Died	306	125,086	92,125	158,047	
Patient Outcome—LTFU					**<0.001**
In care or transferred out	14,211	20,018	17,532	22,505	
LTFU	616	69,882	41,518	98,247	
TB Status					**<0.001**
No TB	14,226	24,090	21,271	26,909	
TB present	343	64,383	41,881	86,884	
WHO Stage					0.09
Stage 1	5026	19,568	15,439	23,698	
Stage 2	3232	25,212	17,729	32,696	
Stage 3	4894	25,826	21,620	30,031	
Stage 4	644	34,336	18,019	50,652	
ART Initiation Mode					**<0.001**
New patient	13,944	25,471	22,555	28,386	
Transferred in	1188	8958	5523	12,392	
Duration under ART in Months					0.35
Less than 3.23 months	91	48,761	-21	97,542	
3.23 to 14.52 months	1804	21,219	15,595	26,842	
14.53 to 40.37 months	5695	22,860	17,982	27,739	
More than 40.37 months	7535	25,571	21,872	29,271	
Province					**<0.001**
Haut-Katanga	4741	8021	5403	10,639	
Kinshasa	10,392	31,541	27,802	35,280	
Rurality/Urbanicity					**<0.001**
Rural	821	3291	1351	5231	
Semi-rural	2038	26,180	18,080	34,281	
Urban	12,274	25,236	22,193	28,279	

Abbreviations: CI, confidence interval; TB, tuberculosis; WHO, World Health Organization; ART, antiretroviral therapy; LTFU, lost to follow-up. Note: Bolded *p*-values indicate the significance of differences at *p* < 0.05.

**Table 3:** Multiple comparisons of mean viral loads from ANOVA for independent variables with >2 categories in HIV/AIDS clinics of Haut-Katanga and Kinshasa provinces, DRC, 2014–2019.

Subgroups		Mean Difference	95% CI		*p*-Value
			Lower	Upper	
Age and Sex					
<15 years, male	<15 years, female	9043	−16,425	34,511	0.92
	15 years or older, male	22,080	1581	42,578	**0.03**
	15 years or older, female	31,636	12,993	50,278	**<** **0.001**
<15 years, female	<15 years, male	−9043	−34,511	16,425	0.92
	15 years or older, male	13,037	−7068	33,142	0.42
	15 years or older, female	22,593	4384	40,801	**<** **0.001**
15 years or older, male	<15 years, male	−22,080	−42,578	−1581	**0.03**
	<15 years, female	−13,037	−33,142	7068	0.42
	15 years or older, female	9556	−547	19,659	0.07
15 years or older, female	<15 years, male	−31,636	−50,278	−12,993	**<** **0.001**
	<15 years, female	−22,593	−40,801	−4384	**0.01**
	15 years or older, male	−9556	−19,659	547	0.07
Rurality/Urbanicity Status					
Rural	Semi-rural	−22,890	−33,038	−12,741	**<** **0.001**
	Urban	−21,945	−26,340	−17,550	**<** **0.001**
Semi-rural	Rural	22,890	12,741	33,038	**<** **0.001**
	Urban	945	−9598	11,487	1.00
Urban	Rural	21,945	17,550	26,340	**<** **0.001**
	Semi-rural	−945	−11,487	9598	1.00

Abbreviations: CI, confidence interval. Note: Bolded *p*-values indicate the significance of differences at *p* < 0.05.

## Data Availability

The program-implementing partners required that data be destroyed after publication. The authors do have data until the publication of the article. The authors can facilitate data access if requested with proper permission from the DRC Ministry of Health.
